# Response of alcohol fermentation strains, mixed fermentation and extremozymes interactions on wine flavor

**DOI:** 10.3389/fmicb.2025.1532539

**Published:** 2025-01-29

**Authors:** Ling-Zhi Zhao, Jing Chen, Xiang-Ying Wei, Bo Lin, Feng-Jin Zheng, Krishan K. Verma, Gan-Lin Chen

**Affiliations:** ^1^College of Light Industry and Food Engineering, Guangxi University, Nanning, China; ^2^Guangxi Subtropical Crops Research Institute, Guangxi Academy of Agricultural Sciences, Nanning, China; ^3^Key Laboratory of Quality and Safety Control for Subtropical Fruit and Vegetable, Ministry of Agriculture and Rural Affairs, Nanning, China; ^4^Guangxi Key Laboratory of Quality and Safety Control for Subtropical Fruits, Nanning, China; ^5^Institute of Agro-Products Processing Science and Technology, Guangxi Academy of Agricultural Sciences, Nanning, China; ^6^Sugarcane Research Institute, Guangxi Academy of Agricultural Sciences, Nanning, China

**Keywords:** alcohol fermentation, bacteria, extremophiles, single and mixed strain fermentation, strain improvement, wine flavor

## Abstract

Winemaking, one of the ancient technologies, is simply the process of converting sugar into alcohol through a complex biochemical reaction. The process of winemaking involves a complex of enological technique that faces a host of challenges in a winery including, inconsistent quality due to chemical and microbiological instability, limited sensory flavor profiles, and concerns met with changing micro-environmental conditions. Fermentation is a metabolic process where the chemical composition of an organic substrate is fragmented via the cellular enzymes under anaerobic conditions. Mixed fermentation, which involves using multiple strains, can enhance the aroma of fermented food, overcome the limitations of single strain fermentation, and improve flavor and quality of food. Mixed fermentation has important applications for agro-food industries, healthcare products and medical sciences. The modern mixed fermentation process showed the enhancement of wine aroma, flavor and taste, reducing volatile acidity and upregulating the phenylethyl acetate concentration through synergistic effect of multiple microorganisms. Key microorganisms in alcohol fermentation, such as yeast, lactic acid and acetic acid bacteria, interact with each other during alcohol fermentation process affects the quality and flavor of the wine. Extremophilic microorganisms have established different molecular strategies to survive amidst the adverse conditions. Biocatalysts isolated by these organisms are termed extremozymes and possess extraordinary properties of salt allowance, thermostability, and cold adaptability. However, the physicochemical and sensory properties of alcohol are important to the quality of end-use products. Therefore, when optimizing fermentation conditions, selecting a right combination of microorganisms is the key to derive better physicochemical and sensory properties. However, the use of mixed fermentation and extremozymes can provide significant insight and potential remedial solutions to overcome these technical problems and shape the final product in more desirable and sustainable ways, challenging the current shortcomings to deliver a more resilient end-products with consistent, flavorful, and a number of what may be considered remedial techniques can be employed to produce an acceptable product to consumers.

## 1 Introduction

Winemaking as a form of food preservation is as old as civilization. The term wine is most commonly used to refer to the fermented product of sugar juice. Similar products can also be obtained from various fruits including apples and pears, dates, bananas, prickly pear cacti, agave, palm, jackfruit and other fruits (Hahn-Hägerdal et al., 2007). In winemaking, the fermentation parameter is the key process where yeast acts as a fermentation enzyme mediating the conversion of sugar (must) in the juice into ethanol (alcohol) and CO_2_. This process is primarily driven by the yeast *Saccharomyces cerevisiae*, which breaks down the sugars, releasing the alcohol and carbon dioxide as byproduct under incomplete oxidation process (Voidarou et al., 2020). Alcoholic fermentation is an essential biochemical process under anaerobic fragmentation of organic compounds by the metabolic processes of microorganisms to release trace amounts of energy. The importance of alcohol is reflected in its wide application in biological and medical sciences to produce food, beverages, energy and medicine (Voidarou et al., 2020; Sun et al., 2022).

In alcoholic fermentation mediated by microorganisms, employing single-strain yeast is usually the mainstream selection in the traditional fermentation industries. Single-strain fermentation has high controllability, and stable fermentation effects can be achieved by precisely controlling fermentation processes to ensure the quantum yield and quality of alcohol (Pinto et al., 2021). However, using a single strain in the fermentation process can simplify the fermentation process and facilitate standardized management and monitoring. Although a single strain has specific advantages, the performance in the fermentation process has certain limitations. For instance, the metabolic pathway of a single strain is relatively simple, and it seldom exert its fermentation potential, resulting in slow fermentation and limited alcohol production capacity (Adebami et al., 2022) perhaps due to attenuation of microbial activities, which are influenced by the fermentation conditions. The fermentation factors such as ambient air temperature, pH regime fluctuates the adaptability of a single strain and eventually affecting the fermentation efficiency. Fermentation parameters with single strain yeast can usually utilize specific sugars, such as glucose or sucrose, with poor conversion capacity to produce complex carbohydrates, like starch or cellulose (Hahn-Hägerdal et al., 2007). Moreover, to meet the high-quality nutrients from single-strain organisms, supplementary ingredients are essentially required to hasten the fermentation process and curtail the production cost to a certain extent. Single-strain organisms are deficient in producing flavor responsive substances and metabolic diversity, and challenging to meet the market demand for diversified by-products.

Given the various shortcomings of single-strain organisms in the fermentation process, current novel strategies with mixed fermentation technology has attracted widespread attention to researchers and the agro-industries (Pettit, 2009). Mixed fermentation is the use of multiple microorganisms in the fermentation process, through the synergy between microorganisms, to pay more attention to the advantages of each strain to improve the fermentation efficiency and product quality (Hoelzle et al., 2014). Mixed fermentation has more robust environmental adaptability than single-strain fermentation and can maintain stable alcohol production in more complex and dynamic fermentation conditions (Reihani and Khosravi-Darani, 2019) to eliminate the risk of stuck fermentation. Moreover, different microorganisms can produce a variety of flavor substances during metabolism, enhancing the flavor and diversity of the products. Mixed fermentation can upregulate the recycling of nutrients to reduce the generation of fermentation waste (Areniello et al., 2023).

During alcohol fermentation process, the types of dominant or latent microorganisms and their synergistic interactions play a crucial role to produce additive quality and flavor of the wine (Yang et al., 2020). Among them, yeast, lactic acid bacteria (LAB) and acetic acid bacteria (AAB) are the key strains in the alcohol fermentation process, and these bacteria interact with each other to combine the fermentation effect and the characteristics of the final products (De Vuyst and Leroy, 2020). Yeast is the main fermentation microorganism in alcohol fermentation, responsible for converting sugar into alcohol and carbon dioxide. The generation of alcohol content, flavor, and aroma complexity can be influenced by the actions of various yeast strains during the fermentation process (Liu et al., 2023). During fermentation, lactic acid bacteria can generate lactic acid, modify the pH of fermentation-related environmental factors, and cease the growth of dangerous microbes (Zapasnik et al., 2022).

Optimal production of lactic acid bacteria (LAB) can enhance the taste and flavor of wine. The interaction between yeast and LAB can create a congenial fermentation environment to promote the production of alcohol and upregulate the diversity of flavor compounds (Watanabe, 2024). In contrast, if the propagules of acetic acid bacteria (AAB) are multiplied in abundant, it leads to production of excessive amount of acetic acid via oxidation, resulting in rise of acidity in the fermentation product by lowering the pH of the fermentation medium, thereby inhibiting optimal yeast proliferation and impeding the fermentation efficiency and masking the natural flavor of the wine (Mendes-Faia, 2020). Therefore, selecting a suitable combination of fermentation bacterial species at right proportions, and providing congenial growing conditions are the key features to improving quality wine production. Curiously, the concomitant combination of physiological, biochemical and sensory properties of alcohol are crucial events of fermentation process (Romero-Rodríguez et al., 2022), to optimize production efficiency, storage stability, shelf life and consumer preference of wine products (Habschied et al., 2022; Tarko et al., 2023).

In addition, some of the extremophilic microorganisms live in adverse conditions also adapt to thrive at varied environmental factors, amidst the ambience extremities with varied temperature regime (55–121°C, and −2 to 20°C), pressure (>500 atmospheres), alkalinity or acidity pH (pH > 8, pH < 4), salinity (2–5 M NaCl or KCl), geological scale/barriers, radiation, chemical changes of heavy metals, nutrients deficiency, such as water, ice, air, rock, or soil, osmotic barriers, or polyextremity (Deppe et al., 2005; Cavicchioli et al., 2011; Dumorne et al., 2017; Patidar and Prakash, 2022). The extremophilic organism *Halothemothrix orenii* serves as a source of a β-glucosidase for possible application in the wine industry. *Halothemothrix orenii* is a true halophilic and thermophilic bacterium whose unique enzymes are described to have broad pH stability and ability to deal with high temperatures and a wide range of salt concentrations (Bhattacharya and Pletschke, 2014).

Many species of extremophiles can also produce a variety of enzymes and biomolecules, including amylase, lipase, xylanase, cellulase, protease, shinorine, mycosporine-like amino acids (MAA), scytonemin, palythine, and porphyra-334 (Habschied et al., 2022; Tarko et al., 2023). Most of these extremophilic enzymes are widely used during different winemaking processes, providing a broad range of beneficial effects, like maximizing juice yield, improving aroma compounds, flavor enhancement, color extraction in red wines, and contributing to the removal of dissolved unwanted colloidal particles and pectin substances during wine stabilization and filtration process (Sarrouh et al., 2012; Espejo, 2021). Extremozymes are generally more capable of withstanding industrial processes in comparison with their mesophilic counterparts (Tarko et al., 2023).

Use of extremophilic enzymes is a novel approach for aroma enhancement in wines. Aroma is considered a key aspect of wine quality. The extremozymes commonly used for traditional food industries are now developed from selected and optimized microorganisms grown at the industrial scale using microbial fermenters strictly under controlled conditions (Espejo, 2021). Consumers demand for premium quality wines providing safe and nutritional characteristics. As with other food industries, winery factories introduce different technological improvements based on novel biotechnology resources, employing dry yeast, acid lactic bacteria starters or enzymes for offering enhanced performance amidst traditionalism of beverage sector (Zhu et al., 2020; Liang et al., 2024). There are different ingredients or additives (potassium sorbate, sulfur dioxide, etc.) and processing aids allowed to be added during production that vary depending on wine type, technological function or country legislation (Lee et al., 2019; Akanbi et al., 2020).

In this context, the use of commercial enzymes in wine industry is related to a variety of goals, especially in the processing (maceration, extraction), stabilization (clarification, filtration) and aging steps (maturation on lees) (Siddiqui et al., 2023; Liang et al., 2024). The reaction potential of such endogenous enzymes should be improved to realize tangible results to produce high-quality wines. This review discusses on state-of-the-art information on the strain improvement, application nuances of efficient strains in alcohol fermentation and optimization of mixed alcohol fermentation process for wholesome production of commercial table wines.

## 2 Modification and optimization of yeast strains

Commercial yeast strains, such as FR yeast, XR yeast, L13 yeast, SP yeast, and LA *Bayanus* yeast are commonly utilized in winemaking (Lee et al., 2019). Most of these yeast strains specifically use a multistage screening process encompassing preliminary, secondary, and tertiary examinations through analysis of aromatic components of fermented mulberry wine (Marullo and Dubourdieu, 2022). In alcohol fermentation, the performance of a single strain is crucial to the fermentation efficiency, product yield, and flavor characteristics. To improve the fermentation efficiency and adaptability of yeast strains, researchers have commonly employed single strain yeasts through a variety of technical processes, such as gene regulation (Brice et al., 2018), physical induction (Yun et al., 2022), chemical mutagenesis (Trovao et al., 2022), natural selection (Rugbjerg and Olsson, 2020) and modern genetic engineering (Eldarov and Mardanov, 2020). Combining these technological approaches can effectively improve the efficiency of the yeast strain during alcohol fermentation.

### 2.1 Natural selection of yeast strains

The selection of yeasts for wine production is usually carried out within the species *Saccharomyces cerevisiae* (Wu et al., 2019). It aims at identifying the yeast strains that besides fermenting juice vigorously and producing high ethanol yield, can also positively influence the composition and the sensorial characteristics of wine. The natural availability of yeast strains possessing an ideal combination of oenological characteristics is highly improbable (Jia et al., 2018). Natural selection is a method of selecting yeast strains with unique characteristics through spontaneous mutation of microorganisms in a natural atmospheric environment without artificial intervention.

Wu et al. (2019) demonstrated that the triphenyltetrazolium chloride (TTC) plates to isolate 209 strains of ethanol-producing yeast from orchard soil and loquat peels. After a series of physical and chemical responses screening and DNA sequencing, strain GP-34 was identified as *Saccharomyces cerevisiae*, which can be used for food fermentation (Wu et al., 2019). Jia et al. (2018) isolated from the orange juice fermentation broth, used Dulbecco's tubule fermentation for primary screening, alcohol production capacity and rough sensory assessment for rescreening, and compared with commercial wine yeast D254 to obtain superior yeast strains. Innovative oenological traits can be introduced or exchanged by hybridizing strains belonging to different species but with a sufficient genetic affinity for them to mate. Interspecific *Saccharomyces* hybrids were found to be stable, vigorous and possessing the parental oenological traits in novel and interesting combinations (Rainieri and Pretorius, 2000).

Molecular biological identification is a method for identifying specific strains of yeast organisms by analyzing their genetic material. The results showed that the strain was associated with the *Saccharomyces cerevisiae* and suitable yeast for the fermentation of orange juice (Jia et al., 2018). The recent development of recombinant DNA technology has overcome the limitations of traditional genetic techniques as well as broadening the potential of wine yeast improvement.

### 2.2 Physical induction of yeast strains

The use of selected starter culture is widely diffused in winemaking. In pure fermentation, the ability of inoculated *Saccharomyces cerevisiae* to suppress the wild microflora is one of the most important features determining the starter ability to dominate the process (Ciani et al., 2016). Since the wine is the result of the interaction of several yeast species and strains, many studies are available on the effect of mixed cultures on the final wine quality (Barrajon et al., 2011). In mixed fermentation the interactions between the different yeasts composing the starter culture can led the stability of the final product and the analytical and aromatic profile. Physical mutagenesis includes traditional UV-mutagenesis technology and new space mutagenesis, such as ion implantation and laser mutagenesis. The mutagenic effect of physical mutagens on species is mainly due to the damage caused by high-energy radiation to organism, which in turn causes a series of gene mutations. Ultrasonic treatment is also often used to improve strains and enhance strain metabolites. Behzadnia et al. (2020) demonstrated the break cell wall of the strain through ultrasonic treatment to promote the absorption and metabolism of nutrients in the strain. Some prior studies have found that the action of ultrasound of appropriate frequency and intensity, the cell wall of yeast cells will be partially damaged, thereby improving the utilization rate of sugar by yeast and promoting the alcohol fermentation rate.

The alcohol fermentation efficiency upregulated by about 20%, and the production of by-products, such as acetaldehyde and acetone was significantly reduced (Yang et al., 2021). The application of atmospheric pressure room temperature plasma (APRT) technology to physically induce mutagenesis of *Clostridium butyricum* to improve the tolerance of wild strains to glycerol (Yun et al., 2022). Microwave mutagenesis combined with *Streptomycin* resistance screening to high-yielding strains of *Virginiamycin* (VGM) (Zhou et al., 2018). Physical induction is more feasible than natural selection, but its unhelpful mutations are more significant than beneficial mutations, making screening process difficult.

### 2.3 Chemical mutagenesis of yeast strains

Chemical mutagenesis is a technique used to improve wine yeasts by introducing random mutations into their genetic code (Jose Moreira Ferreira and Noble, 2019). Chemical mutagens include alkylating agents, natural base analogs, deaminants, frameshift mutagens, hydroxylating agents, and metal salts (Ha et al., 2010). The action of the mutant substance changes the molecular structure of DNA, thereby causing genetic variation. Among them, ethidium bromide (EB) is a commonly used mutagen (Amine et al., 2021). It interferes with the replication and transcription of the strain DNA by embedding into the strain's DNA double strands, causing gene mutations, thereby producing strains with new characteristics (Alnajrani and Alsager, 2020). This approach has been widely used to improve fermentation strains, such as lactic acid bacteria and yeast (Ningthoujam et al., 2024). Some *Saccharomyces cerevisiae* strains tolerate high temperatures and high alcohol concentrations and can ferment quickly after treating *Saccharomyces cerevisiae* strains with mutagens (Amine et al., 2021). These improved strains performed significantly better than untreated strains in alcohol fermentation, specifically in terms of enhanced fermentation efficiency, increased alcohol production, reduced fermentation time, and effective control of the production of byproducts, such as aldehydes and alcohols. Ethylmethane sulfonate (EMS) was applied as a mutagen, and the chemical mutagenesis conditions were determined by its effect on the germination of *Phytophthora sojae*. The newly constructed mutant library laid the genetic material foundation for the functional genomic research of *Phytophthora sojae* (Ha et al., 2010). Similar to physical induction, problems exist, such as more unhelpful mutations than beneficial mutations. Therefore, seeking a convenient and rapid strain optimization method is necessary.

### 2.4 Genetic engineering of strains

In recent decades, many efforts have been made to engineer wine yeast strains with improved characteristics. Genetic engineering is a method of improving production strains by genetic engineering to obtain high-yield engineered strains (Lopez-Malo et al., 2015; Baptista et al., 2021). Genetic transformation can also be used to develop new strains. Baptista et al. (2021) found that *Saccharomyces cerevisiae* can regulate the expression of critical enzymes in the sugar metabolism pathways through gene editing to effectively improve the strain sugar utilization efficiency, thereby increasing alcohol production and reducing the production of by-products. Gene editing can regulate the expression of key enzymes in the sugar metabolism pathways of *Candida*, such as triglycerides and fructose, which can effectively improve the ability to utilize different sugar sources to enhance the efficiency of alcohol production (Chen et al., 2020). Through transcriptomic comparison, Brice et al. (2018) revealed information related to changes in the nitrogen sensing signal system between wine-type and non-wine-type strains. The CRISPR-Cas system obtains yeast strain with increased phenyl ethyl acetate (PEA) production, which can make alcoholic beverages more pinkish and honey-colored (Vigentini et al., 2017).

The emergence of genetic engineering technology has solved the problems of blindness and a large amount of work in previous screening methods. It can specifically transform specific genes to obtain the results according to expectations. Genetic engineering technology has become the most advanced approach for bacterial strain improvement.

## 3 Alcoholic fermentation with mixed bacteria

**Figure 1:**
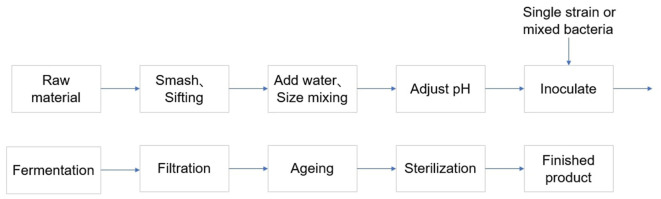
Simplified outline of the mixed bacterial fermentation interaction on the flavor of wine.

In recent years, there is an increasing interest from the winemaking industry for the use of mixed fermentations with *Starmerella bacillaris* (synonym *Candida zemplinina*) and *Saccharomyces cerevisiae*, due to their ability to modulate metabolites of oenological interest (Jacobus et al., 2021). In wine production, lactic acid bacteria are used in a secondary fermentation called malolactic fermentation (MLF) that occurs after the primary alcoholic fermentation (Luyt et al., 2021). With the development of advance fermentation technology, microbial co-cultivation technology has gradually become an important method to improve product yield and diversity (Senne de Oliveira Lino et al., 2021). This method can pay more attention to the advantages of different microbial species by co-cultivating, thereby optimizing the alcohol fermentation process. The application of composite strains is particularly important in alcohol production (Luyt et al., 2021). Microbial co-cultivation technology allows for mutual coordination between different bacterial communities, which helps to overcome the limitations of single strain fermentation processes (Thuan et al., 2022). The interactions of composite strains make it possible to achieve efficient utilization of resources, reduction of waste and synergistic enhancement of metabolites. This method not only improves fermentation efficiency but also may improve the final products flavor and quality, which requires intelligent consumers need for diversification and quality improvement (Figure 1).

*Saccharomyces cerevisiae* is the most widely used strain for alcoholic fermentation and associated in almost all types of alcoholic fermentation, such as wine, beer, liquor and whiskey (Jacobus et al., 2021). Its main function to convert sugars in raw materials fermented into alcohol and carbon dioxide. During the fermentation process, *Saccharomyces cerevisiae* can produce abundant by-products, such as esters, alcohols, aldehydes and organic acids, which influence the aroma and flavor of the fermented wine (Wang et al., 2019). The formation of higher alcohols usually associated two pathways in the fermentation process, involving the degradation metabolic pathway (Ehrlich metabolic mechanism) and the anabolic pathway (Harris metabolic mechanism). Both of these pathways are achieved by the decarboxylation of the corresponding α-keto acid during the fermentation of *S. cerevisiae* (Yang et al., 2019). Likewise, *Candida albicans* plays an important role in some traditional fermentation processes, especially in the brewing of Chinese liquor and rice wine (Wang et al., 2019). Compared with *Saccharomyces cerevisiae, Candida albicans* has some synergistic advantages in fermenting the complex sugars and potential to produce specific aromatic substances to bring unique flavor and taste to fermented products (Chew et al., 2019).

In winery processing, *Bacillus subtilis* is mainly used for solid-state fermentation, especially in producing Chinese liquor, where it can decompose complex starch (Krishna, 2005). This strain can make a large number of enzymes, such as amylase, protease, etc., to decompose complex polysaccharides into simple sugars for yeast to improve fermentation efficiency. However, *Bacillus subtilis* can also produce flavor substances such as amino acids (glycine and alanine), during the fermentation process, which plays a vital role in the flavor formation of liquor (Liu et al., 2024). The genus *Aspergillus* is a filamentous fungus commonly used in solid-state fermentation, especially in traditional Asian fermentation processes, such as producing Chinese liquor and soy sauce (Troiano et al., 2020). A diverse species of *Aspergillus* can secrete various enzymes, such as amylase and protease to decompose macromolecules such as starch and protein in the fermentation of raw materials into simple substances. Therefore, the metabolites of *Aspergillus* also significantly influence the aroma and flavor properties of the final wine products (Zhursinali and Kurmanbaev, 2020).

Among the winery yeast, *Debaryomyces* is a low-temperature and low-water-tolerant yeast species commonly used to ferment wines with specific flavors (Breuer and Harms, 2006). This strain can produce rich esters, giving the fermented wine unique fruity and milky aroma which is especially suitable for making flavored wines or new alcoholic beverages (Angulo et al., 2020). Moreover, the fermentation characteristics of *Debaryomyces* can show good stability under altered environmental conditions.

### 3.1 Microbial interactions in mixed bacterial systems

Mixed fermentations with non-*Saccharomyces* and *Saccharomyces* yeasts benefits wine quality. Interactions among these microbiota influence the wine profile but limited knowledge of these interactions are available. The co-existence of bacteria and yeast is quite common during the alcohol fermentation, and the interactions between mixed bacteria are very complex, mainly combined reactions of yeast-yeast interactions, bacteria-yeast interactions, bacteria-bacteria interactions, filamentous fungi-filamentous fungi interactions, filamentous fungi-bacteria interactions, filamentous fungi-yeast interactions and bacteria-bacteriophage interactions (Sieuwerts et al., 2008; Comitini et al., 2021; Tan et al., 2022).

In the events of the alcohol fermentation, the interactions between different yeast strains significantly affect the fermentation efficiency and flavor. *Saccharomyces cerevisiae* is one of the main yeasts for alcohol fermentation, which can efficiently utilize sugars to produce alcohol (Walker and Stewart, 2016). However, in some high-sugar, high-alcohol fermentation environments, the tolerance of *Saccharomyces cerevisiae* is limited. *Saccharomyces bayanus* has a more robust tolerance to high alcohol and cold, and continue functioning under high-alcohol and low-temperature fermentation conditions (González et al., 2006). When the two coexist, *Saccharomyces bayanus* can compensate for the deficiencies of *Saccharomyces cerevisiae* under these conditions, thereby improving the stability of fermentation and alcohol production (de Melo Pereira et al., 2010). *Aspergillus oryzae* breaks down starch in the early stages of fermentation to produce sugars that yeast can use. Under specific fermentation conditions, *Saccharomyces cerevisiae* strains can produce metabolites, such as glycerol, which help to maintain the osmotic pressure of the fermentation broth and protect *Aspergillus oryzae* from environmental stress (Murado et al., 2008). This synergistic effect of *Aspergillus oryzae* to break down starch more efficiently and provide the sugars needed for fermentation, in which *Saccharomyces cerevisiae* tends to produce alcohol and aroma substances (Fernandez-Gonzalez et al., 2005). The key enzymes play vital roles in the metabolic pathways of aroma compounds during fermentation with *S. cerevisiae* (Yang et al., 2019).

Pectinase yeast has unique advantages in processing raw materials containing pectin, such as fruit fermentation (Fernandez-Gonzalez et al., 2005). Pectinase yeast can degrade pectin and release more fermentable sugars for *Saccharomyces cerevisiae* to increase alcohol production. In this co-fermentation, *Saccharomyces cerevisiae* focuses on alcohol production. At the same time, pectinase yeast improves the utilization efficiency of sugar by breaking down complex carbohydrates, thereby improving the fermentation rate and the quality of final products (Fernandez-Gonzalez et al., 2005). Filamentous fungi can promote fermentation by activating corresponding enzymes to break down complex carbohydrates and release simple sugar that can be used by yeast and bacteria. Moreover, the co-cultivation of filamentous fungi and yeast can improve the aroma compounds of wine to produce the more flavorful products (Zhang et al., 2020; de Castilhos et al., 2023).

### 3.2 Screening for extremophiles

Microbial communities during winemaking are diverse and change throughout the fermentation process. The winery production environments are complex which requires complex of microorganisms including certain specific extremophiles during the process of industrial wine production (Wu et al., 2025). Extremophiles are unique category of microorganisms that can survive and thrive in wider environment, such as extremities of temperatures, pH, salt concentrations and pressures, which are harsher environment for most life forms to withstand (Bosma et al., 2013). Even under the extremities of environmental conditions, extremophilic microbes undergo metabolic pathways that permit to produce corresponding enzymes to active substances (Bosma et al., 2013; Banks et al., 2022). In the recent past, researchers have identified a diverse species of extremophilic microorganisms suited for application in food industries as shown in Table 1.

**Table 1:** Key extremophiles with different action of mechanisms.

**Classification**	**Specie**	**Superiority**	**References**
Thermophilic	*Thermotoga maritima*	α-amylase has the ability to convert starch efficiently at high temperatures.	Santa-Maria et al., 2011
	*Saccharomyces cerevisiae*	It's an excellent ethanol production potential at optimum temperatures.	Nuanpeng et al., 2023
	*Meyerozyma guilliermondii*	It has a very strong ethanol production potential at high temperatures and ability to produce rare sugars.	Nguyen et al., 2022
	*Kluyveromyces marxianus*	Improvement of the quality of whole wheat bread by supplementation of xylanase from *Aspergillus foetidus*	Matsumoto et al., 2018
Psychrophilic	*M. psychrophilia, M. psychrophilia* and *M. robertii*	It shows the ability to ferment wine at low temperatures (10 and 15°C).	Alti-Palacios et al., 2023
	*Mrakia gelida* DBVPG 5952	It has the ability to ferment wine at low temperatures while producing unique volatiles.	De Francesco et al., 2018
Acidophilic	*Issatchenkia orientalis* KMBL 5774	It has the ability to grow under acidic conditions (pH 2.0–3.0) and degrade fumaric acid.	Seo et al., 2007

### 3.3 Role of mixed fermentation process on quality and flavor of fermented products

Mixed fermentation technology achieves an efficient process through synergy and complementary properties between strains. Recent researches in strain improvement for mixed fermentation has gradually developed toward exploiting mixed microbial strains, particular fermentation environments, and high yield and pH tolerance of strains (Lertwattanasakul et al., 2023; Zhao et al., 2024). The method of adding strains and fermentation conditions in the mixed fermentation process are essential factors for determining the quality and flavor of the final wine products (Navarrete-Bolaños and Serrato-Joya, 2023).

Different strains of microbes have significant differences in their environmental requirements during fermentation properties, such as pH, oxygen content, temperature, and nutrient sensitivity. Therefore, it is crucial to reasonably control the time and order of strain addition. Adding yeast in the early stage of fermentation quickly consume sugar for alcohol fermentation, making it difficult for other sugar-dependent microorganisms, such as filamentous fungi, to survive, thus affecting the flavor and product quality of wine (Wei et al., 2022). Developing specific fermentation properties with high-salt environments, anaerobic/aerobic switching, and high-temperature fermentation, can screen out dominant bacterial communities that adapt to these environmental adversities through selective pressure (Wang et al., 2023). Some mixed fermentation systems can accelerate the reaction rate while reducing the risk of contamination when operated at high temperatures. Some screened and genetically modified yeast strains can maintain high activity and strong sugar conversion ability during high-temperature fermentation environments, thereby increasing alcohol production and quality (Caspeta et al., 2015).

High salt or low pH conditions can inhibit the growth of harmful microorganisms while allowing target strains with strong environmental tolerance, such as salt-tolerant yeast or acid-tolerant bacteria, to grow and reproduce in such altered environment (Yin et al., 2019). Mixed fermentation can enhance the tolerance of strains to withstand environmental stress, such as pH, temperature, and oxygen, to increase their product yield through co-cultivation or interaction between microorganisms. Specific lactic acid bacteria (LAB) can effectively upregulate alcohol production by acidifying the fermentation environment, inhibiting the growth of miscellaneous bacteria and providing more favorable environment for yeast (Ewuoso et al., 2020). In liquor fermentation, lactic acid bacteria (LAB) are usually added in the mid and late stages of fermentation process (Zhao et al., 2020). Lactic acid bacteria can inhibit the growth of harmful bacteria by adjusting the pH value and improving the acidity and stability of the wine (Virdis et al., 2021).

**Figure 2:**
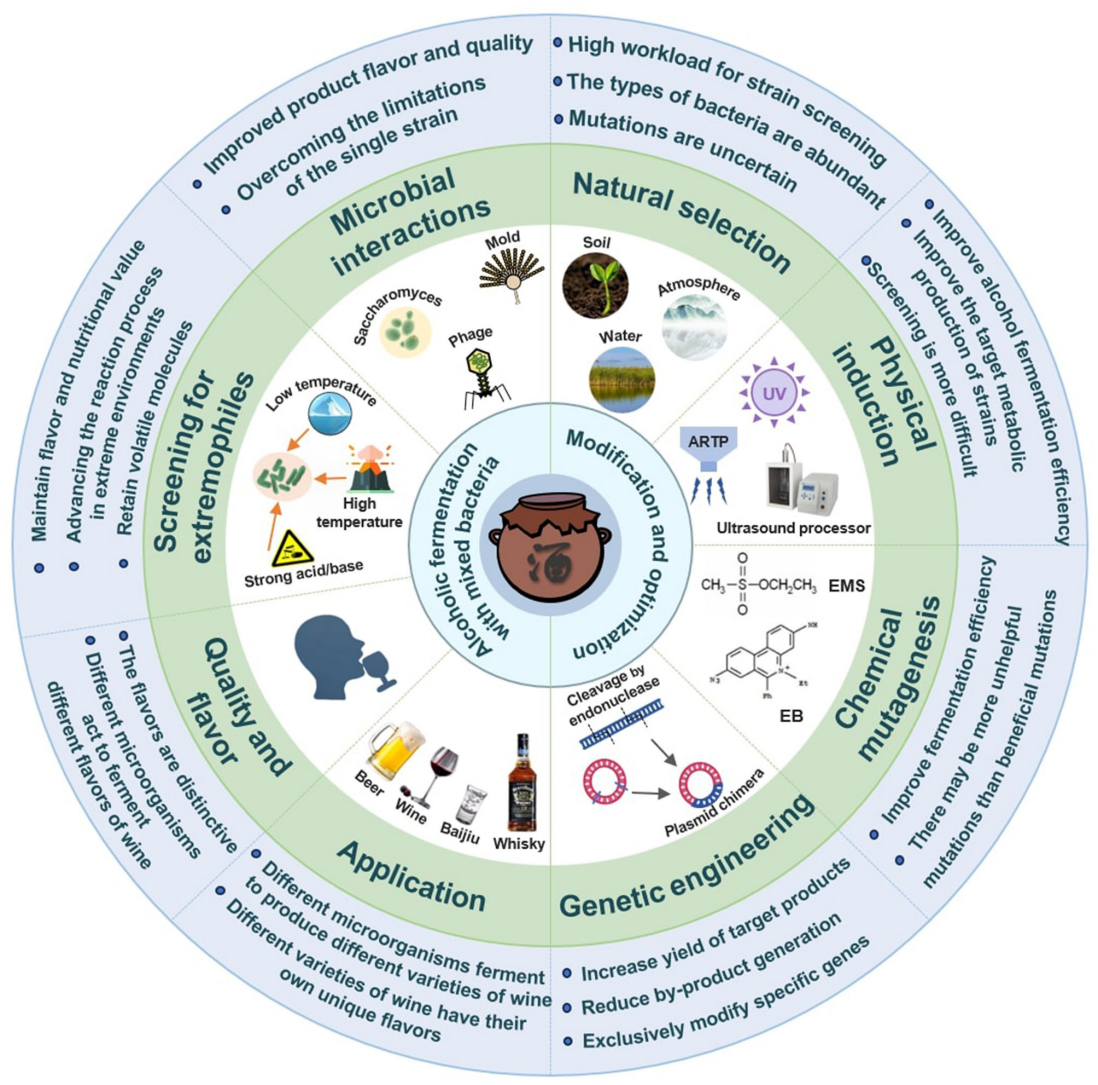
Impact of mixed bacteria during alcoholic fermentation process on wine quality and flavor.

However, adding LAB too early inhibit yeast activity, reducing alcohol production. In some fermentation processes, simultaneous addition of mixed strains of LAB can maximize the synergistic effect between the strains. Similarly, the simultaneous addition of yeast and bacteria can promote each other with enhanced metabolic activities (Viesser et al., 2021). Clearly, the co-fermentation system not only optimizes the pH value of the fermentation liquid through the metabolic activity of lactic acid bacteria but also enhances the efficiency of alcohol fermentation and the diversity of flavor (Walker and Walker, 2018). On the other hand, improper proportions of mixed strains or prevalence of adverse environmental conditions during the alcohol fermentation, however, will lead to excessive growth of particular strain, inhibiting the effects of other beneficial strains and thereby affecting the balance and flavor characteristics of the wine (Figure 2).

### 3.4 Application of mixed-bacteria fermentation technology in wine production

The fermentation process of different wines depends on the activity of microorganisms, and the interaction between these microorganisms directly influences the flavor, quality and stability of the final products (Konig et al., 2009). Whether it is the mutual cooperation between the same microorganisms or competition between different types of microorganisms, these interactions between microorganisms have an essential influence on the formation of wine characteristics (Comitini et al., 2021). In the fermentation process of liquor, mixed fermentation technology is the key process. Liquor fermentation mainly relies on yeast-based alcohol fermentation and the synergistic effect of filamentous fungi and bacteria (Wang et al., 2020). The liquor koji contains a variety of yeasts, lactic acid, acetic acid bacteria and filamentous fungi, which work together in a complex solid-state fermentation environment (Wu et al., 2023). Different yeast strains in liquor form intraspecific synergy through differences in characteristics, such as sugar metabolism ability and tolerance. Some yeast can promote the growth of other yeasts through metabolic intermediates and can even inhibit the growth of harmful microorganisms by producing acid or antioxidant metabolites, such as Rong's yeast and *S. cerevisiae* (González et al., 2006).

The interaction between bacteria and yeast is essential, especially in controlling the acidity of the fermentation environment and stabilizing the fermentation process (Walker and Walker, 2018). Lactic acid bacteria (LAB) can reduce the pH value of the environment and inhibit some unfavorable microorganisms during the fermentation process. At the same time, yeast can better carry out alcohol fermentation with the help of combinations of environmental conditions (Englezos et al., 2019). Filamentous fungi play an important role in the saccharification stage of liquor, providing the sugars required for brewer's yeast fermentation (Chen et al., 2014).

The brewing process of beer mainly relies on the fermentation of maltose and yeast, mainly *Saccharomyces cerevisiae* and *Pasteurella*, its core fermentation bacteria (Willaert, 2012). Compared with liquor, the beer fermentation process is relatively simple, and microorganisms interact less, but it still has a significant effect (Sampaolesi et al., 2023). The synergy between different yeast strains are used to improve the fermentation capacity and flavor of the beer. Yeast affects the fermentation rate, alcohol concentration and aroma production of beer by changing its fermentation ability at different temperatures (Castro et al., 2022). Generally, the growth of bacteria is strictly controlled to avoid affecting the taste of beer. However, specific bacteria are used in a few fermentation processes to provide specific flavor profiles, such as lactic acid bacteria in sour beer (Suzuki et al., 2020).

Wine fermentation depends on wild yeast and artificially inoculated yeast species. *Saccharomyces cerevisiae* is the most common fermentation species (Yilmaz and Gokmen, 2021). In addition, LAB play an essential role in the subsequent malolactic fermentation, especially in red wine fermentation (Virdis et al., 2021). The interaction between different yeast species is often employed to optimize the fermentation performance and flavor generation of wine. Wild yeast usually shows intense activity in the initial fermentation. It is taken over by domesticated artificial yeast to complete the alcohol fermentation process and ensure the efficiency and stability of the fermentation. Lactic acid bacteria play an important role in the malolactic fermentation stage in wine, which can convert the malic acid to lactic acid, thereby balancing the taste. During wine fermentation, the synergistic fermentation of yeast and LAB is the key to improving the stability of wine and the complexity of flavor (Izquierdo-Cañas et al., 2020).

The fermentation of whiskey is similar to that of beer, mainly relying on *Saccharomyces cerevisiae* for alcoholic fermentation (Li et al., 2021). The fermentation process of whiskey is relatively simple, mainly emphasizing the single fermentation of yeast, but the interaction with barrel aging produces the unique flavor of whiskey. In the fermentation of whiskey, different yeast strains are often used to control the fermentation time and flavor, producing different aroma compounds (Waymark and Hill, 2021). Compared with other alcoholic beverages, whiskey has fewer microbial interactions and usually avoids the interactions of other microorganisms to maintain its pure taste.

The fermentation of rice wine is a typical example of multi-species complex fermentation, mainly including yeast, lactic acid bacteria and filamentous fungi (Huang et al., 2024). Similar to Baijiu, rice wine also uses koji for fermentation, and different microorganisms coordinate with each other to complete the saccharification and alcohol fermentation process. Different yeast species in rice wine optimize alcohol production and fermentation stability through mutual metabolic compensation (Zhang et al., 2024), and different yeast species can affect the fermentation frequency and flavor of final products through competition mechanisms. Lactic acid bacteria and filamentous fungi are essential in rice wine fermentation. Filamentous fungi first decompose starch, while lactic acid bacteria adjust acidity in the later stage of fermentation to ensure fermentation stability and taste balance of the final product of wine (Chen et al., 2024).

### 3.5 Extremophiles in fermentation process of wines

The environment of wine fermentation is complex, both on account of the chemical profile and the microbial community, which change over time (Beltran et al., 2001). Enzymes are proteins formed by long chains of amino acids with peptide bonds with particular structures produced by living cells; they are specific biological catalysts involved in different biochemical reactions (Dumorne et al., 2017). Extremophiles have gained more interest owing to their ability to catalyze reactions and potential industrial applications under adverse conditions (Geng et al., 2018; Espejo, 2021). Extremozymes were identified several decades ago; researchers are still focusing on the genetic engineering of existing enzymes to potentiate their activity and the screening of novel enzymes from various sources to obtain the necessary characteristics amenable to industrial and biotechnological applications (Liang et al., 2024).

A lot of research groups and companies around the globe are committed to engineering microorganisms genetically with desirable industrial characteristics suitable for their industrial purposes (Ye et al., 2023). The beneficial effects of fermented foods are mostly developed to bioactive peptide fractions produced during fermentation through microbial protein breakdown (Şanlier et al., 2019). Enzymes from extremophilic microorganisms provide different biotechnological opportunities for biocatalysis and biotransformations due to their stability at high and low temperatures, range of pH, ionic strengths, salinity (Hermann et al., 2018), and the ability to function in organic solvents that would denature most other enzymes (Karmakar and Ray, 2011; Adrio and Demain, 2014; Deshmukh and Jagtap, 2022).

### 3.6 Biochemical properties of extremozymes in wine production

Extremozymes are enzymes that can catalyze chemical reactions in harsh conditions, such as extreme temperatures, pH, and pressure during the events of wine production. Extremozymes are thermophilic enzymes active in harsh environments. These enzymes have a more compact structure with an increased number of hydrophobic interactions, hydrogen bond networks, ion pairs, and disulfide bonds than their counterparts functional in mesophilic organisms. Extremozymes are derived from extremophilic organisms and are capable of catalyzing chemical reactions in harsh conditions (Bleve et al., 2016). They have several biochemical properties that make them useful in wine production, including: Microbial communities during winemaking are diverse and change throughout the fermentation process (Bleve et al., 2016). Microorganisms not only drive alcohol fermentation, flavor and aroma but also enhance wine functional compounds (James et al., 2023). Traditionally, studies on wine microbial interactions have mostly focused on the two key fermentation processes, such as alcoholic fermentation by *Saccharomyces cerevisiae* and malolactic fermentation by *Oenococcus oeni* (Balmaseda et al., 2023).

The contributions of yeast and bacteria to wine fermentation and the technology chosen are crucial factors that influence the overall wine composition. Mostly, wine fermentation is dominated by *S. cerevisiae*. However, some non-*Saccharomyces* yeasts and bacteria actively take part in developing characteristics of unique wine profile (Bleve et al., 2016). Wine contains an array of antioxidants and phenolic compounds, which have shown a variety of health benefits. Nevertheless, wine has probiotic potential as contributed by a pool of microbial consortia during winemaking (Albergaria and Arneborg, 2016). Antioxidants, bacteriocins, pigments, enzymes, and other food components are increasingly being manufactured utilizing food waste as a fermentation substrate (Yang et al., 2022; Siddiqui et al., 2023). Conclusively, extremophilic organisms express various stress-induced proteins that interact with extremozymes to keep them functional during the events of wine production.

## 4 Conclusions and future recommendations

Wine microbes play an important role in the oenological research and industries to innovate and produce wine with enhanced functional components Mostly, wine fermentation is dominated by *S. cerevisiae*. Enzymes have been used empirically for years, especially in diverse food production process. Industrial application of enzymes is widely accepted and well-established in winemaking to improve production processes or product quality. By incorporating extremophiles into the fermentation process, winemakers may enhance the complexity of wine profiles, introducing novel taste characteristics that appeal to adventurous consumers. Mixed-strain bacterial fermentation technology has become an advanced approach to improve fermentation efficiency, optimize flavor, and enhance strain tolerance efficiency. Traditional single-strain fermentation process faces various limitations due to its low raw material utilization and low fermentation efficiency.

In contrast, the application of composite strains has brought breakthrough progress to the winery fermentation process. Through the synergistic effect of different microbial strains, such as yeast, LAB, *Lactobacillus* and filamentous fungi, significant advantages are shown in the fermentation process of alcoholic beverages, such as liquor, beer and wine. These microorganisms can not only complete their metabolic limitations to more stable and efficient fermentation system but also play a key role in enhancing tolerance under specific fermentation environmental variables, thereby effectively increasing alcohol production and improving product quality. With the development of novel biotechnology, the combination of single-strain improvement and multi-strain mixed fermentation is expected to become the mainstream way to improve alcohol fermentation efficiency with product diversity and quality.

Future research should focus on isolating and evaluating specific strains of extremophiles that show promise for additive flavor profiles and assessing their impact on the wholesome wine quality. Collaborations between microbiologists and viticulturists could pave the way for designing innovative approaches that not only improve the sensory attributes of wines but also contribute to establish a more sustainable and create environmentally benign models for future winery industry. As advanced researches continue to evolve, extremophiles are poised to make significant contributions to harness various fields of food industry to ensure the boundless insights leading to potential of life in the extremities of conditions and paving the way for innovations for next generation winery industry. Integration of all essential elements together, wine like other food products should be consumed in an amount beneficial to human health.
